# Clinician perspectives on the utility and acceptability of OxSATS: a qualitative study of a novel structured suicide risk assessment tool

**DOI:** 10.1136/bmjment-2026-302650

**Published:** 2026-08-19

**Authors:** Omar Ouaret Sorr, Howard Ryland

**Affiliations:** 1 Department of Psychiatry, University of Oxford, Oxford, UK

**Keywords:** Psychiatry, Mental Health, Community Mental Health Services, Emergency Services, Psychiatric

## Abstract

**Background:**

Suicide remains a leading preventable cause of death, and patients presenting with self-harm constitute a high-risk group. The Oxford Suicide Assessment Tool for Self-Harm (OxSATS) is a validated model estimating suicide risk. While its statistical performance is established, its clinical utility remains unexplored.

**Objective:**

To understand clinicians’ views on the feasibility, acceptability and potential workflow integration of OxSATS.

**Methods:**

Semistructured interviews with 15 multidisciplinary National Health Service clinicians explored current suicide risk assessment practice, experience with structured tools and views on OxSATS. Participants applied OxSATS to standardised vignettes to simulate real-world decision-making. Interviews were analysed using reflexive thematic analysis.

**Findings:**

Clinicians valued OxSATS for its simplicity, objectivity and percentage-based outputs. OxSATS was viewed as promoting a shared language around suicide risk and supporting assessment consistency. Alignment between OxSATS estimates and clinical judgement was good. However, some clinicians perceived the tool as underestimating risk associated with violent methods. Key implementation barriers included limited scope for wider psychosocial context and potential over-reliance on the tool by inexperienced clinicians. Participants emphasised that OxSATS should complement, not replace, clinical formulation and highlighted the need for clear guidance to support adoption.

**Conclusion:**

OxSATS was viewed as a promising adjunct to suicide risk assessment following self-harm. Addressing concerns around scope, interpretation of percentage risk and integration into wider suicide risk training will be key to successful implementation.

**Clinical implications:**

This study highlights clinicians’ interest in evidence-based adjuncts to suicide risk formulation and supports the use of probability-based models to enhance decision-making.

**WHAT IS ALREADY KNOWN ON THIS TOPIC**

- Suicide risk assessment is inherently challenging and traditional structured tools have been criticised for low predictive validity and oversimplistic categorical labels (e.g., ‘low risk’). Guidelines often advise against use of risk assessment tools, leading to reliance on highly variable unstructured clinical judgement.

**WHAT THIS STUDY ADDS**

- Clinicians found OxSATS straightforward, supportive of structured decision-making and generally aligned with clinical judgement, with a preference for percentage-based risk estimates over categorical labels. Participants raised concerns about potential over-reliance on structured tools, emphasising the need for integration with holistic clinical formulation.

**HOW THIS STUDY MIGHT AFFECT RESEARCH, PRACTICE OR POLICY**

- The findings suggest clinical support for moving away from checklist approaches towards evidence-based tools that complement rather than replace clinical formulation.

## Background

### Suicide: a growing public health crisis

 Suicide remains a leading cause of preventable death, claiming 800 000 lives annually.^1^ In England alone, 16 people on average die by suicide daily and rates have reached their highest since 1999.^2^ Self-harm is a strong predictor of future suicide, associated with a 20-fold increase in 1-year suicide risk.^3^ Individuals who die by suicide often previously presented to hospital following self-harm, representing missed opportunities to intervene.^4^

### Challenges in suicide risk assessment

Suicide risk assessment remains challenging across emergency, psychiatric and community settings due to its low base rate, stochastic nature and reliance on self-report. Current risk assessment practice often combines clinical judgement with structured suicide risk assessment tools, such as the Columbia-Suicide Severity Rating Scale.^5^ Such tools guide systematic exploration of evidence-based risk factors, including ideation, behaviour and psychiatric history, to predict suicide risk.

However, traditional risk tools have been criticised for poor predictive validity, high false-positive rates and potential to detract from clinical judgement.^6^ Previous research concluded that suicide prediction with existing tools remains marginally better than chance, with no improvement over 50 years.^7^

Although clinicians often favour unstructured clinical judgement, its intra- and inter-rater variability is widely recognised. For example, a recent vignette-based study showed highly variable clinician risk estimates using unstructured clinical judgement.^8^

Beyond accuracy concerns, the recommendation that all self-harm patients receive comprehensive psychosocial assessment and gold-standard treatment overlooks the reality of limited healthcare resources. In practice, this ideal is often unmet: a UK multicentre study found that up to 30% of presenting self-harm patients received no psychosocial review.^9^ Improving the accuracy and reliability of suicide risk assessment during this window is therefore critical to target limited resources.

### OxSATS: a novel suicide risk prediction tool

**Figure 1:**
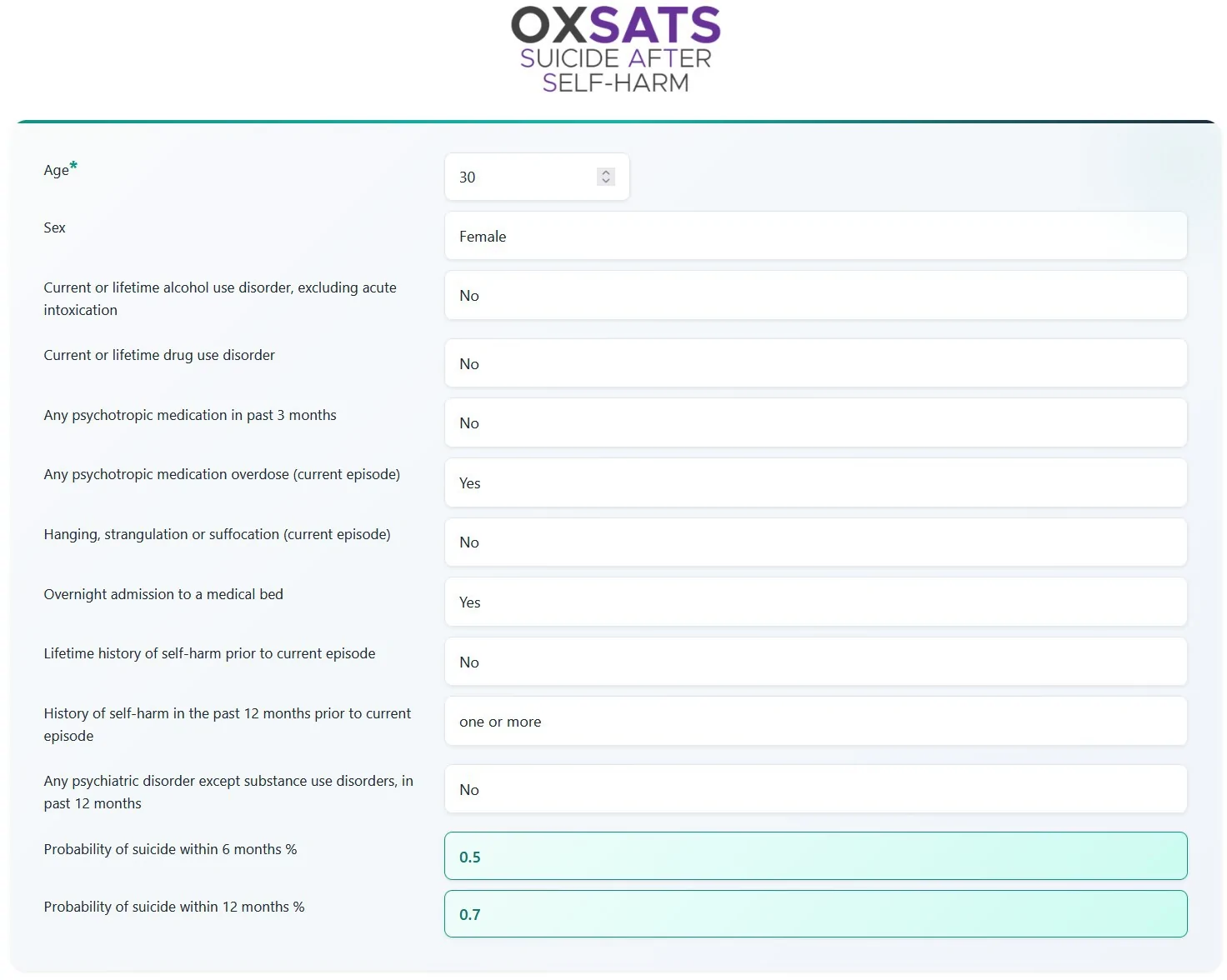
OxSATS risk assessment tool. 11 clinical variables are combined to estimate 6-month and 12-month suicide risk in self-harm patients. Oxrisk.com/OxSATS/. OxSATS, Oxford Suicide Assessment Tool for Self-Harm.

In response to these challenges, the Oxford Suicide Assessment Tool for Self-Harm (OxSATS) was developed as a multivariable prediction model that estimates 6-month and 12-month suicide risk following self-harm. Developed using 53 172 self-harm patients from Swedish linked population registers, OxSATS incorporates 11 sociodemographic and clinical predictors of suicide risk—including psychiatric history, substance misuse and self-harm method (figure 1). This model acts as an adjunct, generating evidence-based risk probabilities that support clinical judgement.

OxSATS was externally validated, transparently reported and developed using robust statistical methods, including calibration. This publicly available web-based tool addresses an important limitation of existing suicide risk tools by presenting the output risk calculation as a percentage, rather than a category, offering a more granular estimate.^10^

### Study rationale

Although the predictive accuracy of structured risk tools has been widely studied, few studies examine their potential or actual clinical application. This study is the first empirical evaluation of the clinical utility of OxSATS, exploring its potential integration into care pathways.

Obtaining such robust evidence is an important step towards implementation,^11^ particularly given recent calls for evaluation of model acceptability and feasibility.^12^ The timeliness of this research is further highlighted by recent National Health Service (NHS) England guidance, which advises against structured suicide risk prediction tools across all mental health settings.^13^

This study also aimed to elicit clinician feedback to optimise OxSATS for real-world use, in line with growing interest in evidence-based tools that support suicide risk formulation and clinical decision-making.

## Objectives

This multisite, multidisciplinary qualitative study aimed to assess the clinical feasibility and acceptability of OxSATS through semistructured clinician interviews. Specific objectives were to (1) explore current approaches and challenges to suicide risk assessment, (2) consider the role of OxSATS within clinical decision-making and (3) identify barriers to the clinical adoption of OxSATS.

This study aimed to identify strengths and limitations of OxSATS, generating actionable insights which could inform the tool’s refinement and potential integration into clinical practice. More broadly, the study sought to contribute to ongoing debates around the utility of structured suicide risk tools and offer clinician-informed perspectives on current challenges in risk assessment.

## Methods

Semistructured interviews explored clinicians’ perspectives on suicide risk assessment and OxSATS. Qualitative methods were employed to capture in-depth, context-specific insights on risk tools and clinical decision-making. Semistructured interviews governed by a topic guide provided consistency to address core research questions across participants while allowing flexibility to explore emergent themes.^14^ Individual interviews were chosen over focus groups to allow for more detailed, candid responses and avoid the influence of group dynamics.

### Topic guide development

The interview topic guide (online supplemental file 1) was informed by the Consolidated Framework for Implementation Research^15^ and followed a three-part structure: (1) general questions on suicide risk assessment, (2) views on structured tools and (3) evaluation of OxSATS. This structure encouraged broader discussions before narrowing the focus.

Topic guide questions were iteratively refined between interviews in response to developing themes and remaining gaps in understanding.^16^ The first three interviews were used as pilots to refine the topic guide (online supplemental file 3).

### Participant recruitment

A multidisciplinary cohort of participants was recruited from NHS trusts across the UK using purposive sampling.^17^ Clinicians were identified via public directories on Trust websites and contacted by email. As data collection and the thematic framework developed, sampling was adapted in line with the constant comparative method.^18^ For example, early under-representation of liaison psychiatry led to targeted recruitment in this specialty.

Participants were qualified doctors or nurses, proficient in English, with experience conducting suicide risk assessments. Specific expertise in suicide prevention was not required to ensure diversity and reflect real-world users. Potential participants were contacted outlining study aims and format; however, the tool itself (OxSATS) was not disclosed prior to interview to minimise bias. Following interviews, participants could recommend colleagues who also met inclusion criteria, allowing snowball sampling to reach suitable participants through peer referral.^19^

In line with best practice, sample size was guided by information power.^16^ The narrowly defined objectives, data-rich 1-hour interviews and inclusion of participants with subject-matter expertise support this approach.

### Data processing and analysis

#### Interviews

Semistructured interviews were conducted using a topic guide (online supplemental file 1) exploring clinician perspectives on suicide risk assessment and OxSATS. To support practical evaluation, participants applied OxSATS to two hypothetical patient vignettes (online supplemental file 2), designed to reflect realistic clinical scenarios which align with OxSATS input parameters. Vignettes differed in demographics and clinical features, as vignette 1 represented a higher risk profile based on OxSATS variables.

Vignettes were used to simulate clinical reasoning in a standardised format, enabling consistent comparison across interviews. This method demonstrates strong concordance with real-world decision-making and is effective in ethically sensitive contexts.^20^ Vignettes were developed iteratively by the authors according to best practice guidelines to optimise clinical realism. HR is an experienced consultant psychiatrist with extensive experience of assessing suicide risk and developing clinical vignettes. Vignettes were tested and further refined in three pilot interviews (online supplemental file 6). Vignettes were presented in a standardised way to all participants as part of a structured interview.

#### Transcription

Zoom audio recordings were transcribed verbatim using OtterAI transcription software. Transcripts were manually reviewed line-by-line and subsequently edited to remove any identifying information.

#### Thematic analysis

Transcripts were imported into NVivo 15 software for systematic data management and underwent qualitative thematic analysis using a six-phase framework.^21^ This involved iterative transcript familiarisation, coding, development of subthemes and synthesis into overarching themes aligned with the research questions. All transcripts were reanalysed against evolving codes to ensure alignment with emerging insights, which also informed iterative refinement of the topic guide.

#### Data presentation

Descriptive statistics were used to summarise clinician-generated suicide risk estimates for each vignette, including arithmetic mean 6-month and 12-month scores. Alignment between OxSATS output and clinical judgement was visualised using bar charts. All quantitative data were processed using Microsoft Excel. Given the small sample size and qualitative design, formal statistical testing of inter-rater variability was not conducted.

## Results

### Results summary

Fifteen clinicians participated in video interviews conducted between January 2025 and February 2026, representing emergency/acute medicine, liaison psychiatry, older adult psychiatry and community mental health specialities (table 1). Approximately 15 hours of dialogue (1 hour per participant) were analysed. Three overarching themes were identified: (1) current approaches and challenges in suicide risk assessment, (2) perceptions of structured risk assessment tools and (3) OxSATS utility and acceptability. See online supplemental file 4 for all subthemes and online supplemental file 7 for representative quotes.

**Table 1:** Summary of relevant participant demographics

Participant characteristics		N
Sex	Male	10
	Female	5
Professional background	Emergency Medicine Doctor	4
	Acute Medicine Doctor	1
	Acute Medicine Nurse	1
	Liaison Psychiatry Doctor	5
	Liaison Mental Health Nurse	1
	Old Age Psychiatry Doctor	1
	Old Age Psychiatry Nurse	1
	Community Mental Health Nurse	1
Experience working in mental health service (years)	0–5	2
	6–10	4
	11–20	5
	>20	4
Location of current practice	Oxford	8
	London	5
	Other UK	2

### Theme 1: current approach and challenges

#### Assessment methods

All participants conducted suicide risk assessments daily, relying largely on unstructured clinical judgement informed by experience, pattern recognition and colleague consultation. No participant routinely used structured tools that generated risk scores. Instead, assessments focused on identifying red flag features, including age, gender, protective factors, behavioural escalation and suicidal ideation. Protective factors such as support networks were also considered; however, the weighting of these factors varied considerably.

#### Speciality-specific considerations

Emergency clinicians prioritised immediate safety and discharge suitability over long-term risk, relying heavily on liaison psychiatry for ambiguous cases. Liaison psychiatrists assessed more severe presentations under time pressure, focusing on current triggers even in patients with repeat presentations. Older adult specialists emphasised longitudinal assessment and rapport-building, given greater stoicism and reluctance to disclose suicidal thoughts in older patients. Oncology and palliative care clinicians highlighted the challenge of distinguishing passive death wishes from active intent near end-of-life. Community mental health teams viewed risk assessment as inherently therapeutic, while managing patient disengagement and variable referral quality from other services.

#### Confidence in assessment

Most participants reported confidence in identifying risk factors but distinguished this from predicting suicide, which was viewed as inherently uncertain. All participants considered risk assessment vital for informing management, care planning and safety decisions. However, some psychiatrists cautioned that emphasis on suicide risk can create assessment tunnel vision, overshadowing broader mental health needs and potentially disengaging patients.

#### Current challenges

Core difficulties in suicide risk assessment included the fluctuating nature of suicidality and rapidly evolving risk factors. Emergency departments were described as non-therapeutic environments which constrained assessment. Conflicting patient information, particularly when patient behaviour contradicted stated intent, posed challenges, especially with older adults and men, who may mask distress. Clinicians reflected on the impact on personal and team morale of attempted or completed suicides following suicide risk assessments. Half of participants, primarily from emergency medicine, reported no formal or recent training in suicide risk assessment, relying instead on knowledge gained at medical school and clinical experience.

### Theme 2: perceptions of structured risk assessment tools

#### Ideal features

Participants strongly agreed on the desirable features of a risk tool: speed of completion, simplicity of use, electronic system integration, use of readily available data and minimised subjectivity through objective inputs. Ideal tools should prompt consideration of core risk and protective factors without constraining clinical judgement. Patient-centred features, such as ease of explanation, were valued by some clinicians.

#### Undesirable features

Tools perceived as lengthy, difficult to complete, reliant on inaccessible information, not integrated into electronic record systems or lacking clinical utility were viewed unfavourably. Difficult to interpret outputs and lack of population-specific validation were also viewed unfavourably.

#### Balancing clinical judgement and structured tools

Participants universally emphasised that clinical judgement must remain central, with structured tools providing guidance to encourage holistic thinking—rather than rigid checklists. Ten participants (66%) expressed concern that such tools could detract from clinical reasoning and prioritise clinician convenience over patient-centred care.

### Theme 3: OxSATS utility and acceptability

#### Perceived strengths

Ten participants (66%) valued OxSATS’ ease of use, describing it as straightforward, user-friendly and quick to complete. The binary yes/no format and routinely accessible input parameters streamlined data entry. OxSATS was viewed as a helpful prompt when considering evidence-based risk factors, particularly in time-pressured or complex cases. The percentage-based output and absence of categorical labels were seen as supporting patient-centred discussions and reflecting the concept that suicide risk is a continuum.

#### Concerns

Nine participants (60%) raised concerns that OxSATS does not capture wider psychosocial predictors of suicide risk such as comorbidities, social support, suicidal ideation, life events and protective factors. Many recommended adding free-text fields for contextual information. Participants identified unclear wording in some input parameters (particularly ‘overnight admission’, ‘psychotropic medication’ and ‘drug use disorder’—online supplemental file 5), raising the possibility of inconsistent tool completion. Participants expressed concerns about the hazards of tools displacing clinical judgement, especially for junior clinicians. Community and liaison psychiatry clinicians emphasised that risk assessment is often itself therapeutic, helping patients feel heard; a benefit potentially undermined by checklist approaches. Commonly proposed safeguards included prompts to consider contextual factors and more explicit disclaimers that OxSATS is an adjunct to clinical assessment that contains non-exhaustive variables.

#### Alignment with clinical judgement

**Figure 2:**
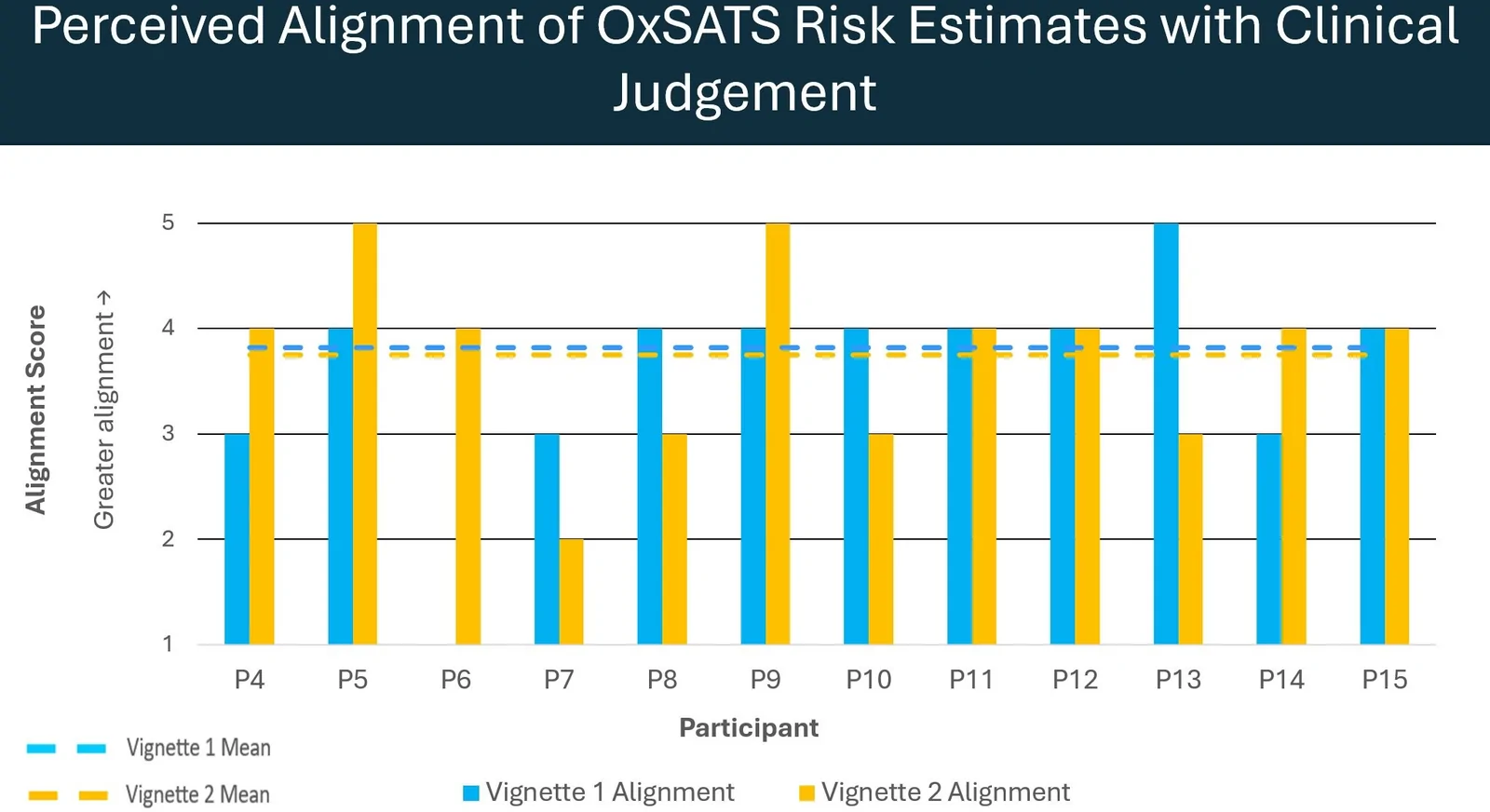
Illustration of clinician-rated alignment scores comparing OxSATS risk estimates with clinical judgement for vignettes 1 and 2. Scores ranged from 1 (completely unaligned) to 5 (perfect alignment). Alignment scores were added after pilot studies P1–P3. P6 did not provide an alignment score for vignette 1. Vignette 1 mean alignment score: 3.82. Vignette 2 mean alignment score: 3.75. OxSATS, Oxford Suicide Assessment Tool for Self-Harm.

All participants completed two vignette-based OxSATS assessments, demonstrating good alignment with professional judgement (mean alignment scores 3.82 and 3.75 for vignette 1 and vignette 2, respectively, figure 2). Clinicians generally agreed with relative risk rankings, though both older adult specialists viewed the lower-scored vignette as more concerning. Over half questioned the OxSATS risk score for the higher-risk vignette, perceiving it as underestimating risk.

#### Risk communication

Eleven participants (73%) preferred percentage-based estimates over categorical labels, viewing percentages as more nuanced and reflective of risk as a continuum. Categorical labels were criticised as misleading and outdated. However, the clinicians emphasised the need for clearer interpretative guidance, noting that defining thresholds of concern presents challenges and over-reliance on numerical output could undermine clinical nuance.

#### Workflow integration

All participants agreed OxSATS should serve as an adjunct to routine practice, rather than as a standalone tool, with a few participants specifying potential roles in early-stage assessment to structure thinking and support formulation. Static input variables were seen to limit longitudinal applicability, as risk outputs may change little for repeated presentations. Participants from emergency settings valued OxSATS as a possible mechanism for prioritising patients for mental health review. Some highlighted the potential for screening out lower-risk patients, and fostering a shared language around risk across services and experience levels.

#### Barriers to adoption

While most participants felt confident using clinical judgement alongside structured tools, senior clinicians felt junior colleagues would likely prefer formal guideline support for such tools. Participants suggested that OxSATS should be embedded within broader suicide risk education and that clinical vignettes would be useful for training. Without direct questioning, six participants spontaneously expressed willingness to trial OxSATS pending further validation.

## Discussion

This study represents the first empirical investigation of OxSATS’ clinical acceptability and feasibility, exploring how multidisciplinary clinicians engage with novel suicide risk assessment tools. Through semistructured interviews with 15 NHS clinicians, using vignette-based assessments, we examined current assessment practices, perceptions of structured tools and OxSATS-specific utility.

Overall, findings suggest that OxSATS is viewed as a clinically useful adjunct that supports more nuanced risk formulation, prompts structured enquiry and encourages clearer communication across services. Clinicians valued its objectivity, simplicity and percentage-based risk estimates over categorical stratification, while emphasising that OxSATS should complement rather than replace clinical formulation. Our results highlight both the potential value and implementation challenges of structured suicide risk assessment tools in contemporary practice.

### Structured tools support, rather than replace, clinical judgement

Clinicians across diverse specialities, most of whom perform daily suicide risk assessments, valued OxSATS for prompting systematic consideration of evidence-based risk factors, particularly in time-pressured or ambiguous cases. Participants also highlighted its potential to support consistency and a shared language of risk, aligning with research demonstrating structured tools improve inter-rater reliability.^22^

Marked variation was observed in how clinicians interpreted identical vignettes, including which risk factors were prioritised, their relative weighting and which patient was deemed higher risk. This reflects wider evidence demonstrating the subjectivity and inconsistency of unstructured clinical judgement,^7^ even in comparable vignette-based studies.^8^

Despite recognising their potential benefits, no participant routinely used structured risk tools comparable to OxSATS in practice, relying instead on assessments shaped largely by patient narratives and psychosocial context. This mirrors the decline in tool use following critiques of predictive validity and increasingly sceptical clinical guidance.^7 23^ A central barrier to adoption was the emphasis placed on professional ownership of assessment. While participants agreed that tools should support rather than replace judgement, concerns were raised about a ‘checklist mentality’, particularly among less experienced clinicians, echoing broader critiques of structured tools as oversimplifying complex clinical realities.^6^ Senior clinicians further emphasised the therapeutic value of assessment itself, reflecting evidence from randomised controlled trials.^24^ Risk tools should therefore aspire to preserve and, where possible, enhance therapeutic engagement.

Despite national consensus moving away from structured tools (largely based on older models not developed using advanced methods), a survey of all 85 NHS mental health organisations found widespread use of risk tools, with 94% of tools producing scores that inform clinical management.^25^ Furthermore, almost half of this study’s participants spontaneously expressed interest in trialling OxSATS in clinical practice.

Several clinicians distinguished between prediction and formulation: while suicide was viewed as inherently difficult to predict, OxSATS’ percentage output was valued as an objective aid to formulation rather than a binding forecast. Many participants adopted a pragmatic stance, treating the probabilistic estimates as prompts for reflection rather than definitive truth, supporting the need for hybrid assessment models that integrate validated risk models within formulation-led clinical decision-making.

### Preference for quantitative output, but with contextualisation

Clinicians generally favoured percentage estimates over categorical labels such as ‘low-medium-high’ risk, viewing percentages as more nuanced reflections of a risk continuum, consistent with National Institute for Health and Care Excellence guidance that categorical labels convey a false sense of certainty.^26^ Despite this, participants emphasised the need for contextual guidance to interpret percentage outputs meaningfully. One improvement introduced after pilot studies was using analogies such as: ‘if you were to review 100 patients with this OxSATS risk profile, X would be predicted to die by suicide within 6 months’, which was well-received.

Determining actionable risk cut-offs was also challenging. While no consensus emerged, clinicians supported training that encourages local, context-sensitive thresholds based on service capacity, population and risk tolerance. Ultimately, any thresholds should be regularly reviewed, remain holistic and address need irrespective of risk score.

### Workflow integration: context matters

OxSATS’ perceived utility was highly context-dependent. Most clinicians preferred OxSATS for early-stage assessments or as a structured ‘sense-check’ in borderline cases. Some cautioned against its longitudinal use given its relatively static variables.

Differences in preferred use likely reflected varying assessment goals across specialities: for example, emergency clinicians, prioritising acute safety, found OxSATS’ 6-month to 12-month risk horizon less relevant, whereas community psychiatry adopted a more longitudinal view. These findings reinforce calls for context-sensitive risk tools^27^ and extend this premise by identifying setting-specific use cases, including the potential value of speciality-adapted OxSATS versions, for example, an emergency department variant estimating risk within a shorter timeframe.

### Challenges to OxSATS adoption and areas for improvement

**Figure 3:**
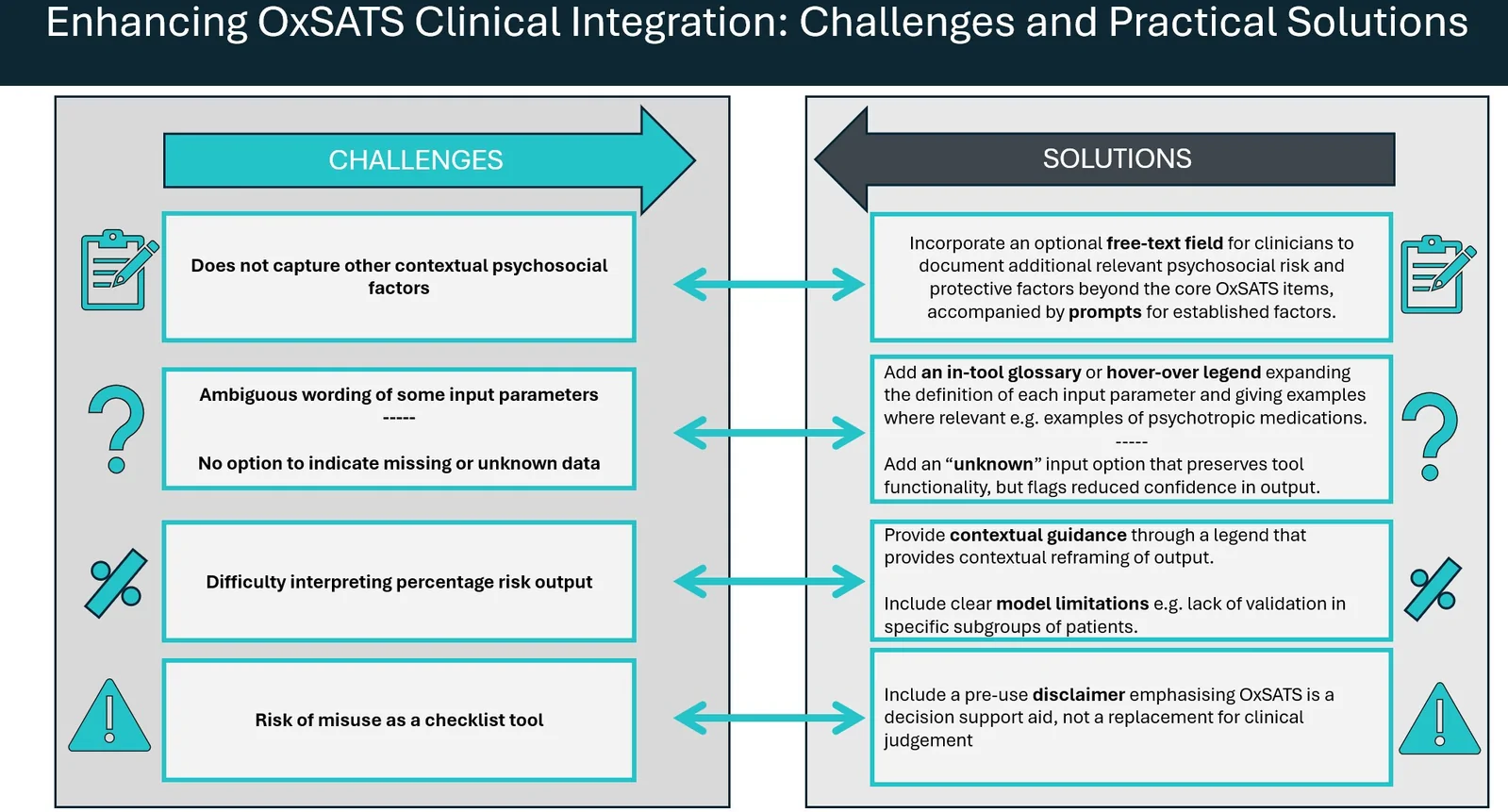
Key barriers and potential solutions to support OxSATS integration into clinical practice. OxSATS, Oxford Suicide Assessment Tool for Self-Harm.

While OxSATS was broadly welcomed, participants raised noteworthy concerns (see figure 3). The most consistent critique was its limited ability to capture psychosocial context. Some clinicians reported ambiguity about how OxSATS should be weighted alongside clinical formulation, highlighting the importance of clearly defining tool scope.

Furthermore, although a composite outcome of self-harm and suicide may increase statistical power, these outcomes use distinct predictors and have different management implications. This was explored through OxSATS’ sister self-harm model OxSET,^28^ which validated repeat self-harm prediction against a composite outcome of repeat non-fatal self-harm and suicide, with model performance remaining essentially unchanged. OxSET may therefore be complementary to OxSATS rather than a justification for combining both scores.

Initial concerns were raised about potential over-reliance on numeric risk estimates, echoing sentiments that probabilistic tools may encourage reductionist thinking and could be misused if risk output is treated as objective truth rather than part of broader clinical formulation.

However, empirical findings from this study demonstrate a clear and consistent distinction between suicide risk and clinical need, particularly among psychiatrists, who emphasised that probabilistic estimates should not be conflated with decisions about access to care and that a relatively low OxSATS score does not imply low distress or reduced need for intervention. When discrepancies arose between clinical judgement and OxSATS output, clinicians described prioritising judgement and using higher-than-expected OxSATS output as prompts for reflection and re-evaluation. This aligns with recent methodological commentary calling for empirical evaluation of how probabilistic models are understood and used in practice^11 12^ and suggests that risks associated with tool use are already recognised and actively managed by clinicians.

Another key insight was that approximately a third of participants—particularly emergency clinicians—reported no formal postgraduate suicide risk training despite frequent exposure to high-risk cases. This highlights an opportunity to embed OxSATS within broader training frameworks while reinforcing best practice, including holistic and needs-based assessment.

Taken together, this suggests successful OxSATS implementation requires more than the tool alone. It points to the need for a holistic strategy that clarifies tool scope, safeguards against over-reliance and integrates training to enhance wider clinical assessment and decision-making.

### Strengths

Notably, this study is the first to empirically examine the feasibility and acceptability of OxSATS, focusing on end-users to directly address a recognised gap in suicide prevention research where structured tools are often developed without systematic clinician input.^29^ The sample was multidisciplinary, combining decades of clinical experience across five specialities. The inclusion of nursing professions, often under-represented, further broadened the clinical relevance and transferability of findings. The interviewer’s non-specialist background in suicide research likely reduced interpretive bias and facilitated candid participant reflection.

Methodologically, the study followed qualitative best practice, including iterative topic guide refinement (online supplemental file 6), reflexivity and attention to construct and content validity. Standardised vignettes enabled comparative exploration of OxSATS and highlighted variability in unstructured clinical judgement.

### Limitations

Several limitations should be acknowledged. First, the seniority of most participants may limit generalisability to junior clinicians, whose perspectives and training needs may differ. Although the sample size (n=15) was appropriate for qualitative depth and information power, it did not permit statistical generalisation or subgroup analysis, for example, analysis of inter-rater variability.

Furthermore, the absence of general practitioners in the sample limits transferability of findings to primary care, where suicide risk is often first identified.^30^ While patient perspectives were raised by participants, direct patient involvement was beyond this study’s scope and represents an important priority for future research. Finally, although coding was conducted iteratively with supervisory oversight, independent double-coding would strengthen credibility.

### Clinical implications

These findings suggest structured tools such as OxSATS are most accepted in practice when explicitly positioned as adjuncts to clinical formulation. Clinicians used OxSATS to structure thinking, prompt consideration of evidence-based risk factors and support clearer interdisciplinary communication, particularly in early or ambiguous assessments, suggesting an unmet need. The strong preference for percentage-based output over categorical labels further supports a move away from reductive risk stratification, provided numerical estimates are accompanied by clear interpretative guidance.

Short-term, OxSATS should be refined using this study’s recommendations to improve usability and confidence in risk outputs, including clarifying input definitions and developing guidance for interpreting percentage estimates. Broader stakeholder engagement, including resident doctors and primary care professionals, would promote acceptability across pathways. Longer-term, real-world implementation studies should evaluate OxSATS’ impact on decision-making, interservice communication and patient trajectories. While cluster randomised trials may offer rigorous evaluation, the low base rate of suicide and difficulties in capturing nuanced clinical practice highlight the value of mixed-method approaches. Further work should assess cost-effectiveness, subgroup validity and locally defined intervention pathways. Embedding OxSATS within broader training on suicide risk formulation could support hybrid approaches that integrate structured assessment with clinical judgement.

## Supplementary material

10.1136/bmjment-2026-302650 online supplemental file 1

## Data Availability

Data are available upon reasonable request.
