# Supplementary material for: Clinician perspectives on the utility and acceptability of OxSATS: a qualitative study of a novel structured suicide risk assessment tool
Source: BMJ Ment Health. 2026 Aug 19;29(1):e302650. doi: 10.1136/bmjment-2026-302650 (PMC13504839; doi:10.1136/bmjment-2026-302650)
Supplement: Supplementary file 1 [file bmjment-29-1-s001.docx]

Supplementary Materials

## Supplementary 1 - Semi-Structured Interview Topic Guide

Note: not all questions were asked in every interview. All participants were asked questions relating to core research objectives, for example perceived OxSATS’ strengths and concerns. Additional questions were explored adaptively, considering participant role, experience and themes that emerged during the allotted interview time.

**Introduction**

- Thank participant for taking the time to interview
- Briefly summarise researcher background and study aims/objectives:
  - Exploration of suicide risk assessment tools
  - Over the next 30-60 minutes, we will explore your thoughts on this tool from an on-the-ground, clinical perspective, rather than purely academic
  - There is not an expectation of expertise in suicide risk assessment
  - Interview is structured into four phases:
    - 1.) General questions about suicide risk assessment
    - 2.) Perspectives on structured tools
    - 3.) Practical evaluation of OxSATS using clinical vignettes
    - 4.) Discussion of OxSATS potential utility, acceptability, advantages, disadvantages and barriers to adoption
- Confirm participant understands that the interview will be audio recorded, transcribed and anonymised and the purpose of this data for a Masters’ dissertation and potential future publication
- Check if participant has any questions before commencing the interview

Participant professional background

- Ask participant to state their professional background, including speciality and number of years’ experience in mental health and any other relevant roles beyond their clinical role e.g. mental health research lead

General Questions About Suicide Risk Assessment

- How frequently do you assess suicide risk?
- How important is suicide risk assessment in your service?
- What is your approach to assessing suicide risk?
- How confident do you feel in assessing suicide risk?
- What training have you received in suicide risk assessment?
- What challenges do you face when assessing suicide risk in your setting?

Questions About Structured Risk Assessment Tools

- Have you used any structured tools for suicide risk assessment?
- What features of a structured assessment tool would be helpful in your practice?
- What features would discourage you from using such a tool?

Clinical Vignettes

You will now be shown short vignettes that represent hypothetical clinical scenarios simulating real-life situations. Each vignette includes a patient history, clinical presentation, and key risk factors. After review, I will ask you to provide:

- Your overall clinical judgment
- Risk rating
- OxSATS to calculate a risk score

<https://oxrisk.com/oxsats/>

******Vignettes shared via screen share during the interview, giving participant time to read in their own time**

**Vignette 1:**

Patient S, aged 25, arrives at A&E accompanied by her neighbour following an attempted hanging. She appears drowsy and reports intentionally overdosing on recently prescribed Clonazepam three days ago. She experiences frequent hopelessness and says "It would be easier if I wasn't here"

- Diagnosis: Borderline personality disorder and major depressive disorder
- History: Multiple self-harm incidents in past 12 months, including hospitalisation for overdose three months ago
- Social context: Unemployed, minimal social support, lives alone, facing possible eviction due to missed rent

On a scale of 1-5, how aligned is the OxSATS risk % score with your clinical judgement for this patient?

- 1.) Completely unaligned: The % score completely differs from my clinical judgement
- 2.) Slightly aligned: There is some overlap, but a large discrepancy exists between OxSATS and my clinical judgement
- 3.) Moderately aligned: The risk % partially aligns with my clinical judgement, but there is a notable difference
- 4.) Mostly aligned: The % score closely matches my clinical judgement, with only a minor difference
- 5.) Perfectly aligned: The % score fully and accurately reflects my clinical judgement

**Vignette 2:**

Patient J, aged 45, presents to the Emergency Department with non-cardiac chest pain. During psychosocial assessment, he discloses suicidal thoughts. He also has fresh superficial cuts on his forearm that he says he intentionally caused.

- Diagnosis: Generalised anxiety disorder
- History: Weekly alcohol binge drinking, inconsistent sertraline adherence over the past 6 months, no prior episodes of self-harm
- Social context: Financial distress, undergoing divorce

On a scale of 1-5, how aligned is the OxSATS risk % score with your clinical judgement for this patient?

- 1.) Completely unaligned: The % score completely differs from my clinical judgement
- 2.) Slightly aligned: There is some overlap, but a large discrepancy exists between OxSATS and my clinical judgement
- 3.) Moderately aligned: The risk % partially aligns with my clinical judgement, but there is a notable difference
- 4.) Mostly aligned: The % score closely matches my clinical judgement, with only a minor difference
- 5.) Perfectly aligned: The % score fully and accurately reflects my clinical judgement

Questions About OxSATS

Acceptability and Feasibility

- What are your overall impressions of OxSATS?
- What are your initial biggest concerns about using this tool?

Input Data Availability

- How readily available are the required OxSATS input parameters in your clinical setting?
- Which parameters are typically difficult to obtain or missing?
- How often must practitioners estimate or make assumptions about input parameters?

Time Requirements

- What is the typical time needed to gather OxSATS input data?
- How long does it take to interpret and act on the results?

Workflow Integration

- How well does OxSATS integrate with existing suicide risk assessment workflows?
- At what points in the patient care pathway would OxSATS be most and least valuable? E.g. at first assessment or after psychological review?
- What changes to current procedures would OxSATS require?
- What barriers might affect OxSATS adoption in clinical practice?

Output Data

Risk Output

- Which risk presentation format do practitioners prefer (percentages, categories, or other)?
- How does the presentation format influence interpretation and clinical decisions and which do you prefer?
  - Rephrase this by giving example using vignette e.g. vignette 1, how would they interpret and act on ‘high risk’ output vs ‘X% output’

Risk Perception

- How do practitioners distinguish between high, medium, and low risk levels?
- How do practitioners weigh OxSATS output against clinical judgment?

Impact on Practice

Clinical Decision-Making

- How might OxSATS output influence patient care decisions (e.g., referrals, follow-ups)? Would it make a difference to management?
- Would OxSATS affect confidence in clinical judgment?
- Could OxSATS lead to over-reliance on or underuse of clinical judgment?

Ethics

- Might OxSATS affect service access for low-risk patients?
- What is the best way to communicate OxSATS results to patients and families?

Fit and Role Within Service

Implementation Frameworks

- Are practitioners familiar with NICE guidelines on predictive tools?
- How does the absence of NICE endorsement affect OxSATS perception?
- What would large-scale implementation require?

Purpose and Value

- Do you think OxSATS would be more effective at identifying high-risk patients, screening out low-risk patients, or both?
- How relevant are technical metrics (e.g. sensitivity, specificity, positive predictive value, negative predictive value, calibration) to practitioners?
- How does OxSATS compare with other risk assessment tools? Complements or conflicts with existing tools?

Usability

- How intuitive and user-friendly is the tool for practitioners?
- What aspects of the design or operation could be improved?
- How confident are clinicians in data entry and output interpretation?

Feedback

- What is the best way to incorporate feedback for OxSATS improvement?

Training

- What training resources would best support OxSATS implementation?

Conclusion

Ask the participant if they have any final points they wish to get across based on our discussion or beyond or if they are happy to conclude.

Thank participant for their time and offer to share OxSATS model development research paper.

## Supplementary 2 - Patient Vignettes

*Appendix 2: Fictional patient vignettes presented during interviews. Vignette 1 incorporates higher-risk OxSATS parameters. Vignettes were shared with participants via screen share.*

## Supplementary 3 - Research Methods

*Appendix 3: visual summary of key methodological steps, from participant recruitment to theme analysis.*

## Supplementary 4 - Thematic Summary

*Appendix 4: Summary of final themes, subthemes and corresponding key insights. Suicide Risk Assessment (SRA).*

## Supplementary 5 - Proposed Fixes to OxSATS wording

*Appendix 5: Summary of OxSATS input parameters clinicians found unclear, with proposed wording improvements.*

## Supplementary 6 - Topic and Vignette Guide Refinement Log

List of changes made to the topic guide or vignette following each interview

- P1 (pilot):
  - Participant needed clarification on input parameter clarity, especially overnight admission and lifetime history of substance abuse. Subsequent interviews will prioritise questions in wording clarity
- P2 (pilot):
  - Required clarification that OxSATS is an adjunct and not a standalone tool. Topic guide updated to reflect this, with introduction emphasising the supportive nature of the tool. Subsequent questions to include a prompt on the difference between risk formulation and risk prediction.
- P3 (pilot):
  - Marked variation noted in participants’ description of alignment between clinical judgement and OxSATS rating, 1-5 alignment scale added to subsequent interviews following vignette evaluation.
  - Vignette 1 modification: changed Xanax into Clonazepam in **vignette** 1 to align with UK prescription practices.
- P4
  - Participant struggled to interpret percentage-based risk output. Analogy-based framing of risk introduced following vignette completion by participants
- P5
  - Participant requested examples of “barriers to implementation”, leading to greater clarification of what this alludes to in future questions by giving examples if participants struggle to understand the question
- P6
  - Highlighted context-specific considerations regarding ED discharge acute safety considerations. Topic guide amended to focus more on context-specific uses and challenges
- P7
  - Participant noted the use of technical metric (positive predictive value), potential need to prompt future clinicians about the importance of technical metrics
- P8
  - Also reinforced the importance of probing for context-specific use scenarios.
- P9
  - Clearly differentiated between risk formulation vs prediction. Topic guide updated to re-emphasise this distinction in subsequent interviews
- P10
  - Mentioned the importance of OxSATS training using vignettes but also practicing its use without over-formalising training. Topic guide amended to include question probing the balance between over and under-training the tool
- P11
  - Notion of clinical assessment itself being a therapeutic intervention central to this interview. Topic guide updated to probe future clinician agreement with this concept
- P12
  - Participant emphasises the need to ensure that OxSATS risk outputs are not used to exclude patients from receiving care. Topic guide refined to probe the separation between suicide risk and clinical need
- P13
  - Participant mentioned the use of a tool that outlines patient risk and how those needs will be correspondingly mitigated, but no use of a risk scoring tool such as OxSATS. Once again mentions the issues with clarity of wording of certain input variables e.g. admission to medical bed
- P14
  - Clinician highlighted the importance of cultural appropriateness of any tools used, particularly in diverse settings such as central London. Also emphasised the adjunctive role of OxSATS
- P15
  - The ability to clearly interpret risk output scores and provide clinicians with recommended practice based on a given output was deemed particularly important

### Supplementary 7 - Representative Quotes
